# Short-term clinical outcomes of 6-Fr versus 8-Fr catheters for percutaneous transhepatic gallbladder drainage in acute calculous cholecystitis: a propensity score-matched analysis

**DOI:** 10.1186/s12876-026-05210-9

**Published:** 2026-08-06

**Authors:** Tetsushi Azami, Yuichi Takano, Naoki Tamai, Jun Noda, Fumitaka Niiya, Masatsugu Nagahama

**Affiliations:** Department of Medicine, Division of Gastroenterology, Showa Medical University Fujigaoka Hospital, Yokohama, 227-8501 Japan

**Keywords:** Percutaneous transhepatic gallbladder drainage, Acute cholecystitis, Percutaneous cholecystostomy, Catheter size

## Abstract

**Background:**

Percutaneous transhepatic gallbladder drainage (PTGBD) is a standard drainage option for patients with acute cholecystitis at high surgical risk. The association of between catheter diameter and clinical outcomes remains unclear. We compared short-term clinical outcomes between 6-Fr and 8-Fr catheters used for PTGBD.

**Methods:**

This single-center retrospective observational study included patients who underwent PTGBD for acute calculous cholecystitis. Baseline characteristics between the 6-Fr and 8-Fr groups were adjusted using propensity score matching (PSM). Outcomes were assessed for adverse events (AEs), technical success, clinical success, and 30-day mortality.

**Results:**

A total of 127 and 149 patients were included in the 6-Fr and 8-Fr groups, respectively. After PSM, 71 matched pairs were analyzed. After matching, the AE rate was significantly higher in the 6-Fr group than in the 8-Fr group (28.2% vs. 9.9%; risk difference [RD] 0.18; relative risk 2.85; *p* = 0.009). Clinical success was significantly higher in the 8-Fr group at days 5 and 7 (91.5% vs. 100%; RD = − 0.08; *p* = 0.041) but was similar between the groups by day 14 (98.6% vs. 100%; *p* > 0.999). No significant between-group differences were observed in technical success or 30-day mortality.

**Conclusions:**

Compared with 6-Fr catheters, 8-Fr catheters were associated with fewer short-term AEs and may be associated with earlier clinical improvement. An 8-Fr catheter may be a reasonable option for short-term PTGBD management.

**Supplementary Information:**

The online version contains supplementary material available at https://doi.org/10.1186/s12876-026-05210-9.

## Background

Percutaneous transhepatic gallbladder drainage (PTGBD) is a standard procedure for patients with acute cholecystitis who are at high surgical risk [1]. After it was first reported in 1979 [2], subsequent studies demonstrated a stable clinical success rate, ranging from 85.6% to 97.5% [3–5].

Recently, alternative drainage techniques, such as endoscopic transpapillary gallbladder drainage and endoscopic ultrasound-guided gallbladder drainage, have been developed. However, these procedures require advanced endoscopic expertise and are available only at a limited number of centers [6, 7]. Therefore, PTGBD remains the mainstay for managing acute cholecystitis [1].

The PTGBD procedure is relatively standardized and achieves a high technical success rate [8–10]. The Society of Interventional Radiology has proposed a benchmark technical success rate of 97.9% [11].

Nonetheless, evidence regarding device-related factors remains limited, and an optimal catheter size has not been established. In clinical practice, catheters ranging from 6- to 10-Fr are generally used [1], with selection largely dependent on operator preference and patient characteristics. Larger-diameter catheters may provide more efficient drainage but may be associated with increased liver parenchymal injury and bleeding risk. Conversely, smaller-diameter catheters are often considered less invasive but carry a higher risk of insufficient drainage, catheter occlusion, and dislodgement. Nevertheless, data on the trade-off between efficacy and safety remain scarce.

At our institution, 6–8-Fr catheters are used primarily according to clinical indications. This study aimed to evaluate differences in clinical outcomes and safety between 6-Fr and 8-Fr catheters.

## Methods

### Study design and participants

This was a single-center retrospective study. The participants’ characteristics were retrospectively reviewed using their medical records. Patients who underwent PTGBD for acute calculous cholecystitis at Showa Medical University Fujigaoka Hospital between January 1, 2017, and August 31, 2025, were included if relevant clinical data were available.

The inclusion criteria were (1) PTGBD performed for acute calculous cholecystitis, and (2) clinical and procedural data available in the medical records. The exclusion criteria were (1) concomitant gallbladder cancer; (2) subsequent surgical cholecystectomy performed within 5 days after PTGBD; (3) use of a catheter size other than 6-Fr or 8-Fr during the initial PTGBD; and (4) death within 24 h after PTGBD when the cause of death was judged to be unrelated to the procedure.

The study was conducted in accordance with the principles of the Declaration of Helsinki. The SHOWA Medical University Research Ethics Review Board approved the study protocol (approval number: 2026-0033). The requirement for informed consent was waived because anonymized clinical data were used, and an opt-out approach was applied.

### Outcomes and definitions

Acute cholecystitis was diagnosed and its severity graded according to the Tokyo Guidelines 2018 [1]. Chronic kidney disease (CKD) was defined as a documented history of CKD or an estimated glomerular filtration rate < 60 mL/min/1.73 m². Chronic liver disease was defined as a history of chronic liver disease or imaging findings suggestive of liver surface irregularities.

Preprocedural computed tomography (CT) was used to assess gallbladder morphology and the surrounding anatomy. The short-axis diameter of the gallbladder was measured on an axial image to capture the maximum short-axis dimension. Gallbladder-liver apposition was evaluated on the same axial image as the proportion of the gallbladder wall in contact with the liver and categorized into four groups: 0–25% (Category 1), 25–50% (Category 2), 50–75% (Category 3), and 75–100% (Category 4). The presence of ascites was determined based on CT findings. All imaging assessments were performed independently by two physicians, and discrepancies were resolved by consensus.

The primary outcome was the overall incidence of adverse events (AEs) following PTGBD. AEs were evaluated according to the Clavien–Dindo classification [12] and encompassed events occurring within 30 days after PTGBD. All patients were followed for 30 days after PTGBD using inpatient, outpatient, and readmission records. Secondary outcomes were specific AEs, technical success, clinical success, and 30-day mortality. Specific AEs included catheter dislodgement, catheter occlusion, bleeding, and bile leakage. Catheter dislodgement was defined as either complete displacement of the catheter from the gallbladder or partial migration resulting in loss of the appropriate intracholecystic position of the catheter tip and requiring catheter exchange. Minor positional changes that did not impair catheter function and required no intervention were not classified as catheter dislodgement. Catheter occlusion was defined as inadequate drainage requiring flushing, catheter exchange, or re-intervention.

Technical success was defined as the successful placement of the catheter within the gallbladder. Clinical success was defined as improvement in fever and abdominal pain, with normalization of the white blood cell count, within 5 days after PTGBD. Clinical success was assessed by investigators who were not blinded to catheter size. Cumulative clinical success was also assessed by days 7 and 14 using the same criteria.

As an additional exploratory analysis, subsequent clinical courses after PTGBD, including subsequent cholecystectomy, catheter management, and recurrent cholecystitis after catheter removal, were retrospectively assessed in the propensity score-matched cohort.

### Procedure details

All PTGBD procedures were performed through a transhepatic route using the Seldinger technique by a team of two gastroenterologists (an operator and an assistant), at least one of whom specialized in pancreatobiliary interventions. Catheter size was selected at the operator’s discretion.

Before puncture, 15 mg of pentazocine was administered intravenously for analgesia, and lidocaine was infiltrated at the puncture site. Color Doppler imaging was used to confirm the absence of intervening vessels along the planned puncture route. The gallbladder was then punctured under ultrasound guidance using an 18-gauge needle (Hanaco Medical, Saitama, Japan). A small amount of bile was aspirated to confirm intraluminal access.

A 0.035-inch guidewire (Radifocus; Terumo, Tokyo, Japan) was advanced into the gallbladder under fluoroscopic guidance. After a small skin incision and tract dilation, a pigtail catheter (UreSil L.L.C., Illinois, USA) was inserted.

Catheter position was assessed using plain abdominal radiography on the day after placement, with additional radiographic evaluations performed as clinically indicated.

### Statistical analysis

Continuous variables are presented as medians (interquartile ranges), and categorical variables are presented as numbers (percentages).

Propensity score matching (PSM) was performed to adjust for baseline imbalances between the two groups (6-Fr vs. 8-Fr). Covariates were selected based on their clinical relevance and availability at the time of catheter-size selection. Propensity scores were estimated using a logistic regression model that included age, sex, American Society of Anesthesiologists Physical Status (ASA-PS), hematologic disease, chronic liver disease, CKD, antithrombotic therapy, severity of cholecystitis, platelet count, gallbladder short-axis diameter, gallbladder-liver apposition, and ascites.

Matching was performed using 1:1 nearest-neighbor matching without replacement, with a caliper width of 0.2 standard deviations of the logit of the propensity score. Covariate balance before and after matching was assessed primarily using the standardized mean difference (SMD), and an SMD < 0.2 was considered indicative of acceptable balance. Before matching, between-group comparisons were conducted using the Mann–Whitney U test for continuous variables and Fisher’s exact test for categorical variables. After matching, paired comparisons were performed using the Wilcoxon signed-rank test for continuous variables and McNemar’s test for categorical variables.

All outcomes were analyzed as binary variables. Before matching, outcomes were compared with Fisher’s exact test. After matching, treatment effects were estimated while accounting for the matched-pair structure, and effect sizes were reported as risk difference (RD) and relative risk (RR) with 95% confidence intervals (CIs). p-values were calculated using McNemar’s test and were considered supplementary.

As a sensitivity analysis, inverse probability of treatment weighting (IPTW) was used to estimate the average treatment effect in the overall cohort. Weighted generalized linear models were fitted to estimate RD and RR, and robust variance estimation (sandwich estimator) was applied to address potential variance underestimation attributable to weighting, thereby providing 95% CIs and p-values.

Post-hoc power for individual AEs was calculated using the continuity-corrected McNemar test for matched binary outcomes at a two-sided significance level of 0.05. E-values were calculated for the point estimates and the CI limits closest to the null for the overall AE rate and clinical success. Post-hoc exploratory subgroup analyses of clinical success stratified by baseline fever and abdominal pain were performed in the PSM cohort using Fisher’s exact test because stratification did not preserve the matched-pair structure. Analyses of individual AEs and subgroups were exploratory, and no adjustment for multiple comparisons was applied.

Missing data were handled using complete-case analysis, and patients with missing covariates required for propensity score estimation were excluded. Statistical significance was defined as a two-sided p-value < 0.05. All analyses were performed using R software (version 4.5.3; R Foundation for Statistical Computing, Vienna, Austria).

## Results

### Participants

**Fig. 1:**
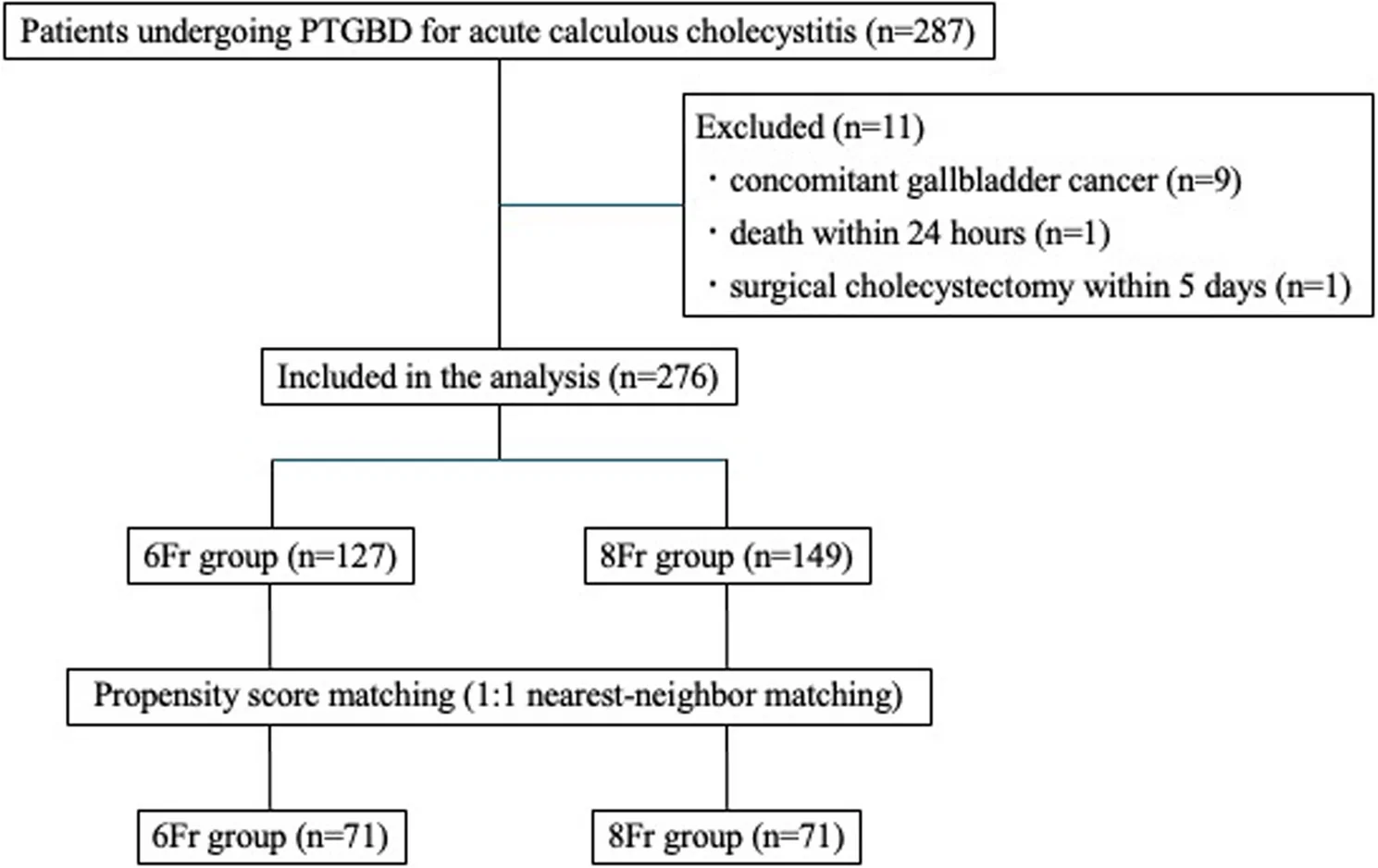
Patient flow diagram. Flowchart of patient selection and cohort construction. Among 287 patients who underwent percutaneous transhepatic gallbladder drainage (PTGBD) for acute calculous cholecystitis, 11 were excluded (concomitant gallbladder cancer, n = 9; death within 24 h, n = 1; cholecystectomy within 5 days, n = 1). Overall, 276 patients were included in the analysis (6-Fr, n = 127; 8-Fr, n = 149). After propensity score matching (1:1 nearest-neighbor matching), 71 matched pairs were obtained

During the study period, 287 patients underwent PTGBD for acute calculous cholecystitis. After excluding nine patients with concomitant gallbladder cancer, one patient who died within 24 h, and one patient who underwent cholecystectomy within 5 days, 276 patients were included in the analysis. Among these, 127 and 149 were assigned to the 6-Fr and 8-Fr groups, respectively. PSM (1:1 nearest-neighbor matching) yielded a matched cohort of 71 patients in each group (*n* = 142) (Fig. 1). Thirty-day follow-up was complete for all patients. PTGBD procedures were performed by 26 primary operators with varying levels of clinical experience, including physicians in their third postgraduate year.

### Descriptive data

Before matching, baseline characteristics differed between the two groups, including the Charlson Comorbidity Index, ASA-PS, antithrombotic therapy, severity of cholecystitis, abdominal pain, and platelet count (Table 1). A substantial imbalance was evident for antithrombotic therapy (56.7% vs. 16.1%, SMD = 0.93) and ASA-PS (SMD = 0.487). After PSM, covariate balance improved for most variables, with SMDs generally < 0.2. Nevertheless, mild residual imbalances remained for fever (SMD = 0.287) and abdominal pain (SMD = 0.214).

**Table 1:** Baseline characteristics before and after propensity score matching

	Pre-matched cohort (*n* = 276)				Matched cohort (71 pairs)			
	6-Fr group (*n* = 127)	8-Fr group (*n* = 149)	*p*-value	SMD	6-Fr group (*n* = 71)	8-Fr group (*n* = 71)	*p*-value	SMD
Age, years	76 [69.5, 83.0]	74 [67.0, 82.0]	0.207	0.177	76 [69.0, 83.0]	78 [70.0, 82.5]	0.650	0.028
Male sex	88 (69.3)	88 (59.1)	0.081	0.215	47 (66.2)	48 (67.6)	0.596	0.030
Body mass index, kg/m²	21.8 [18.7, 24.1]	22.4 [19.8, 24.7]	0.132	0.158	22.0 [20.2, 24.3]	21.6 [19.8, 23.5]	0.877	0.118
Charlson Comorbidity Index	6.5 [5.0, 8.0]	5.0 [4.0, 7.0]	< 0.001	0.425	6.0 [4.0, 8.0]	6.0 [4.0, 8.0]	0.446	0.005
ASA-PS	3.0 [2.0, 3.0]	2.0 [2.0, 3.0]	< 0.001	0.487	3.0 [2.0, 3.0]	3.0 [2.0, 3.5]	0.241	0.074
Chronic kidney disease	25 (19.7)	27 (18.1)	0.759	0.040	11 (15.5)	14 (19.7)	> 0.999	0.111
Hematological disease	6 (4.7)	3 (2.0)	0.31	0.151	2 (2.8)	3 (4.2)	> 0.999	0.076
Chronic liver disease	12 (9.4)	12 (8.1)	0.831	0.049	7 (9.9)	7 (9.9)	0.789	< 0.001
Antithrombotic therapy	72 (56.7)	24 (16.1)	< 0.001	0.930	23 (32.4)	23 (32.4)	> 0.999	< 0.001
Severity of acute cholecystitis			0.01	0.400			> 0.999	< 0.001
Nonsevere	107 (84.3)	143 (96.0)			66 (93.0)	66 (93.0)		
Severe	20 (15.7)	6 (4.0)			5 (7.0)	5 (7.0)		
Concomitant common bile duct stones	16 (12.6)	18 (12.1)	> 0.999	0.016	6 (8.5)	6 (8.5)	0.803	< 0.001
Fever	67 (52.8)	59 (39.6)	0.3	0.266	36 (50.7)	26 (36.6)	0.223	0.287
Abdominal pain	107 (84.3)	138 (93.9)	0.017	0.294	60 (84.5)	64 (91.4)	> 0.999	0.214
White blood cell count, /µL	14110.1 [10395.0, 18495.0]	13840.2 [9950.3, 18090.5]	0.97	0.021	13100.2 [10385.1, 18875.3]	13550.0 [9555.3, 18050.4]	0.909	0.005
Platelet count, ×10⁴/µL	18.2 [13.3, 23.8]	21.8 [16.3, 27.0]	0.001	0.380	20.5 [15.6, 26.1]	20.9 [15.4, 25.2]	0.434	0.008
Gallbladder short-axis diameter, mm	42.0 [35.0, 48.5]	40.0 [37.0, 46.0]	0.868	0.020	41.0 [34.5, 49.0]	40.0 [36.0, 47.5]	0.392	0.038
Gallbladder-liver apposition			0.539	0.176			0.452	0.190
Category 1	12 (9.4)	12 (8.1)			8 (11.3)	7 (9.9)		
Category 2	65 (51.2)	89 (59.7)			37 (52.1)	42 (59.2)		
Category 3	45 (35.4)	44 (29.5)			25 (35.2)	20 (28.2)		
Category 4	5 (3.9)	4 (2.7)			1 (1.4)	2 (2.8)		
Ascites	10 (7.9)	23 (15.4)	0.063	0.237	10 (14.1)	9 (12.7)	> 0.999	0.041

Values are presented as medians [interquartile ranges] for continuous variables and counts (percentages) for categorical variables

*SMD* Standardized mean difference, *ASA-PS* American Society of Anesthesiologists Physical Status

### Outcome data

Clinical outcomes in the unmatched and propensity score-matched cohorts are summarized in Table 2. In the matched cohort, the overall AE rate was significantly higher in the 6-Fr group than in the 8-Fr group (20/71 [28.2%] vs. 7/71 [9.9%]; RD 0.18 [95% CI 0.06–0.30]; RR 2.85 [1.33–6.25]; *p* = 0.009). Regarding specific AEs, catheter dislodgement occurred in 14/71 patients (19.7%) in the 6-Fr group and 7/71 patients (9.9%) in the 8-Fr group, with a higher incidence in the 6-Fr group without reaching statistical significance (RR 2.00 [0.89–4.55]; *p* = 0.146). In the 6-Fr group, four of the 14 patients had partial catheter dislodgement requiring catheter exchange, and ten had complete catheter dislodgement. In the 8-Fr group, two of the seven patients had partial catheter dislodgement requiring catheter exchange, and five had complete catheter dislodgement. Partial catheter dislodgement was managed by catheter exchange and classified as Clavien–Dindo grade IIIa, whereas complete catheter dislodgement was managed with continued antibiotic therapy without repeat gallbladder drainage and classified as grade I. No patient developed bile leakage, biliary peritonitis, or other complications related to catheter dislodgement. Following these management strategies, acute cholecystitis improved in all patients, and none required emergency cholecystectomy. Catheter occlusion occurred in 3/71 patients (4.2%) in the 6-Fr group and in none of the patients in the 8-Fr group (*p* = 0.248). Of these three cases, two resolved with catheter flushing and were classified as Clavien–Dindo grade I, whereas one required catheter exchange and was classified as grade IIIa. Bleeding occurred in 1/71 patient (1.4%) in the 6-Fr group and in none in the 8-Fr group (*p* > 0.999). The bleeding resolved with observation alone and was classified as grade (I) No bile leakage was observed in either group. Two puncture-site infections occurred in the 6-Fr group and were included in the overall AE assessment; both were treated with additional antibiotics and classified as grade (II) Overall, the 6-Fr group had 13 grade I, 2 grade II, and 5 grade IIIa AEs, whereas the 8-Fr group had 5 grade I and 2 grade IIIa AEs. No grade IIIb or higher AEs occurred. The post-hoc statistical power was 29.5% for catheter dislodgement and 8.0% for catheter occlusion and was negligible for bleeding, indicating limited power for comparisons of individual AEs. Power could not be estimated for bile leakage because no events occurred in either group.

**Table 2:** Clinical outcomes before and after propensity score matching

	Pre-matched cohort (*n* = 276)			Matched cohort (71 pairs)				
	6-Fr group (*n* = 127)	8-Fr group (*n* = 149)	*p*-value	6-Fr group (*n* = 71)	8-Fr group (*n* = 71)	RD (95% CI)	RR (95% CI)	*p*-value
Adverse events overall, % (n)	31 (24.4)	13 (8.7)	< 0.001	20 (28.2)	7 (9.9)	+ 0.18 (0.06, 0.30)	2.85 (1.33, 6.25)	**0.009**
Catheter dislodgement, n (%)	22 (17.3)	11 (7.4)	0.015	14 (19.7)	7 (9.9)	+ 0.10 (− 0.01, 0.21)	2.00 (0.89, 4.55)	0.146
Catheter occlusion, n (%)	3 (2.4)	0	0.096	3 (4.2)	0	+ 0.04 (− 0.01, 0.09)	NE	0.248
Bleeding, n (%)	1 (0.8)	1 (0.7)	> 0.999	1 (1.4)	0	+ 0.01 (− 0.01, 0.04)	NE	> 0.999
Bile leakage, n (%)	3 (2.4)	1 (0.7)	0.337	0	0	0.00 (0.00, 0.00)	NE	> 0.999
Technical success, n (%)	127 (100)	149 (100)	> 0.999	71 (100)	71 (100)	0.00 (0.00, 0.00)	NE	> 0.999
Clinical success, n (%)	119 (93.7)	146 (98.6)	0.048	65 (91.5)	71 (100.0)	−0.08 (− 0.15, − 0.02)	0.92 (0.85, 0.98)	**0.041**
30-day mortality, n (%)	2 (1.6)	2 (1.3)	> 0.999	1 (1.4)	2 (2.8)	−0.01 (− 0.06, 0.03)	0.50 (0.05, 5.56)	> 0.999

*RD* Risk difference, *RR* Relative risk, *CI* Confidence interval, *NE* Not estimable due to zero events in one or both groups

Technical success was achieved in all patients in both groups. By day 5, clinical success was significantly lower in the 6-Fr group than in the 8-Fr group (65/71 [91.5%] vs. 71/71 [100%]; RD -0.08 [-0.15 to -0.02]; RR 0.92 [0.85–0.98]; *p* = 0.041). By day 7, cumulative clinical success was achieved in 65/71 patients (91.5%) in the 6-Fr group and 71/71 patients (100%) in the 8-Fr group (*p* = 0.041). By day 14, the corresponding rates were 70/71 (98.6%) and 71/71 (100%), respectively (*p* > 0.999). The between-group difference observed at day 5 persisted at day 7 but was no longer statistically significant at day 14.

There was no significant difference in 30-day mortality between the groups (1/71 [1.4%] vs. 2/71 [2.8%]; RR 0.50 [0.05–5.56]; *p* > 0.999).

The E-values for the point estimate and the confidence interval limit closest to the null were 5.15 and 1.99, respectively, for the overall AE rate and 1.39 and 1.16, respectively, for clinical success.

Subsequent clinical courses after PTGBD are summarized descriptively in Supplementary Table 1.

### Sensitivity and subgroup analyses

Sensitivity analysis was conducted using IPTW. After excluding two patients with missing covariates, 274 patients (6-Fr: 125; 8-Fr: 149) were included in the analysis. The weighted pseudo-population comprised 269.9 and 275.5 patients in the 6-Fr and 8-Fr groups, respectively. The effective sample sizes after weighting were 89.8 and 91.9 in the 6-Fr and 8-Fr groups, respectively. Covariate balance improved across most variables after weighting, although a mild residual imbalance persisted for abdominal pain (SMD = 0.222) (Table 3). In the IPTW analysis, the overall AE rate remained significantly higher in the 6-Fr group (RR: 3.70 [95% CI, 1.85–7.14]). Catheter dislodgement was also significantly more frequent in the 6-Fr group (RR: 3.45 [95% CI, 1.64–7.14]), directionally consistent with the primary analysis. The clinical success rate was significantly lower in the 6-Fr catheter group (RR: 0.93 [95% CI, 0.87–0.98]). Conversely, no significant differences were observed in catheter occlusion, bleeding, or 30-day mortality (Table 4). Overall, the IPTW estimates were directionally consistent with those of the primary analysis.

**Table 3:** Baseline characteristics after inverse probability of treatment weighting

	6-Fr group (weighted)	8-Fr group (weighted)	*p*-value	SMD
Number of patients (pseudo-population)	269.9	275.5		
Age, years	75.0 [68.3, 82.0]	76.9 [68.0, 83.4]	0.645	0.074
Male sex	184.3 (68.3)	170.6 (61.9)	0.382	0.134
Body mass index, kg/m²	21.9 [19.6, 24.2]	21.5 [19.1, 23.9]	0.526	0.115
Charlson Comorbidity Index	6.0 [4.0, 7.5]	6.0 [4.0, 7.0]	0.939	0.023
ASA-PS	3.0 [2.0, 3.0]	3.0 [2.0, 3.0]	0.987	0.013
Chronic kidney disease	43.5 (16.1)	47.9 (17.4)	0.797	0.034
Hematological disease	8.0 (3.0)	6.0 (2.2)	0.668	0.051
Chronic liver disease	24.8 (9.2)	24.0 (8.7)	0.910	0.015
Antithrombotic therapy	94.1 (34.9)	96.2 (34.9)	0.993	0.001
Severity of acute cholecystitis			0.849	0.036
Nonsevere	245.5 (91.0)	247.7 (89.9)		
Severe	24.4 (9.0)	27.8 (10.1)		
Concomitant common bile duct stones	43.1 (16.0)	29.8 (10.8)	0.296	0.151
Fever	125.6 (46.5)	108.1 (39.2)	0.331	0.148
Abdominal pain	237.1 (88.2)	257.3 (94.4)	0.076	0.222
White blood cell count, /µL	13488.7 [10269.3, 18875.1]	13550.0 [9691.5, 18637.1]	0.971	0.004
Platelet count, ×10⁴/µL	20.1 [13.7, 25.6]	20.1 [14.6, 25.1]	0.913	0.056
Gallbladder short-axis diameter, mm	42.0 [35.0, 50.0]	40.0 [37.0, 48.0]	0.807	0.015
Gallbladder-liver apposition			0.983	0.055
Category 1	23.8 (8.8)	22.8 (8.3)		
Category 2	155.3 (57.5)	160.8 (58.4)		
Category 3	81.4 (30.2)	84.7 (30.7)		
Category 4	9.4 (3.5)	7.2 (2.6)		
Ascites	24.7 (9.1)	28.8 (10.4)	0.764	0.044

Values are presented as weighted medians [interquartile ranges] for continuous variables and weighted counts (percentages) for categorical variables, based on the inverse probability of treatment weighting pseudo-population

*SMD* Standardized mean difference, *ASA-PS* American Society of Anesthesiologists Physical Status

**Table 4:** Clinical outcomes after inverse probability of treatment weighting

	IPTW-adjusted RD (95% CI)	IPTW-adjusted RR (95% CI)	*p*-value
Adverse events overall, % (*n*)	+ 0.19 (0.08, 0.28)	3.70 (1.85, 7.14)	**< 0.001**
Catheter dislodgement, n (%)	+ 0.13 (0.05, 0.22)	3.45 (1.64, 7.14)	**0.003**
Catheter occlusion, n (%)	+ 0.03 (− 0.01, 0.07)	NE	0.105
Bleeding, n (%)	0.00 (− 0.02, 0.01)	0.82 (0.05, 12.5)	0.889
Bile leakage, n (%)	+ 0.01 (− 0.01, 0.02)	3.45 (0.36, 33.3)	0.282
Technical success, n (%)	0.00 (0.00, 0.00)	NE	> 0.999
Clinical success, n (%)	−0.08 (− 0.13, − 0.02)	0.93 (0.87, 0.98)	**0.007**
30-day mortality, n (%)	-0.01 (-0.05, 0.04)	1.37 (0.16, 11.1)	0.779

IPTW-adjusted risk differences and relative risks with 95% confidence intervals were estimated using weighted generalized linear models with robust variance estimations. P-values were derived using the same model

*IPTW* Inverse probability of treatment weighting, *RD* Risk difference, *RR* Relative risk, *CI* Confidence interval, *NE* Not estimable due to zero events in one or both groups

In post-hoc exploratory subgroup analyses, clinical success rates in the 6-Fr and 8-Fr groups were 94.1% and 100%, respectively, among patients with fever (*p* = 0.503), 89.2% and 100% among those without fever (*p* = 0.036), 90.6% and 100% among those with abdominal pain (*p* = 0.013), and 100% in both groups among those without abdominal pain (*p* > 0.999).

## Discussion

In this study, we evaluated the association between catheter diameter and clinical outcomes in patients undergoing PTGBD for acute calculous cholecystitis. The overall AE rate was significantly higher in the 6-Fr group than in the 8-Fr group after PSM. Clinical success was lower in the 6-Fr group at days 5 and 7; however, the absolute difference was modest and was no longer observed by day 14. Among individual AEs, catheter dislodgement was numerically more frequent in the 6-Fr group, whereas catheter occlusion and bleeding were uncommon; therefore, definitive between-group comparisons of individual AEs were not possible. Technical success was achieved in all patients, and 30-day mortality did not differ significantly between the groups. The IPTW analysis yielded directionally consistent estimates, although the finding for clinical success should be interpreted cautiously.

The lower overall AE rate observed with the 8-Fr catheter may be attributable to two main factors: drainage efficiency and catheter stability. First, in acute cholecystitis, viscous material, such as purulent bile and debris, often accumulates within the gallbladder. Therefore, a larger lumen is more likely to enable more effective drainage. Second, smaller-diameter catheters are generally less rigid and may be more susceptible to respiratory motion and changes in gallbladder position, potentially increasing the risk of catheter dislodgement. This issue may be particularly relevant in PTGBD, which is performed via a transhepatic route and is influenced by both hepatic and gallbladder movement. Together, these factors may have contributed to the lower AE rates observed with the 8-Fr catheter.

The lower clinical success rates at days 5 and 7 in the 6-Fr group may partly reflect drainage efficiency. Although catheter occlusion occurred only in the 6-Fr group, the small number of events precluded a definitive between-group comparison. Previous studies of percutaneous transhepatic biliary drainage have suggested that larger-diameter catheters may provide more effective drainage [13, 14]; however, these findings cannot be directly extrapolated to 6-Fr versus 8-Fr catheters used for PTGBD. Because drainage performance and catheter stability were not directly measured in the present study, these mechanisms remain hypothetical.

Exploratory subgroup analyses suggested that baseline fever and abdominal pain did not fully explain the lower clinical success rates in the 6-Fr group. However, clinical success was nearly identical between the groups by day 14, suggesting that the observed differences at days 5 and 7 may reflect delayed clinical improvement rather than lower overall treatment effectiveness. Nevertheless, the modest absolute difference, relatively low E-values for clinical success, and small sample sizes within the subgroups indicate that residual or unmeasured confounding cannot be excluded. Therefore, the clinical success findings should be interpreted cautiously.

Although catheter size could theoretically affect procedural difficulty, technical success was achieved in all patients in both groups, indicating that catheter diameter did not affect procedural feasibility. This suggests that use of an 8-Fr catheter did not increase technical difficulty to a clinically meaningful extent under the conditions of this study.

Previous studies have established the clinical utility of PTGBD for acute cholecystitis, with most focusing on overall outcomes or comparisons with alternative drainage techniques [3, 15–17]. In contrast, relatively few studies have examined procedural or device-related factors in detail. Catheter diameter may be an important determinant of both drainage efficiency and catheter stability; however, its clinical impact has not been well characterized. To our knowledge, studies specifically evaluating catheter size in PTGBD remain limited, and this study provides additional insights into this issue.

As shown in the unmatched cohort (Table 1), operators tended to choose smaller (6-Fr) catheters for patients with a higher bleeding risk (e.g., those receiving antithrombotic therapy) or a poorer general condition (higher ASA-PS scores), likely reflecting an attempt to minimize procedural invasiveness. Although it remains unclear whether this tendency can be generalized beyond our institution, it may reflect a common clinical preference. However, after adjustment using PSM and IPTW, the presumed safety advantage of the 6-Fr catheter was not confirmed. The 6-Fr catheter was associated with a higher overall AE rate and lower clinical success. Catheter dislodgement was numerically more frequent in the 6-Fr group in the PSM cohort and was significantly more frequent in the IPTW analysis; however, the post-hoc power for the matched comparison was only 29.5%. Because the individual-AE analyses were underpowered and were not adjusted for multiple comparisons, they should be regarded as exploratory and should not be interpreted as establishing a reduction in any specific AE. Therefore, the present findings do not support the assumption that smaller catheters are safer. Overall, these findings suggest that an 8-Fr catheter may be a reasonable option for short-term PTGBD management. The potential utility of larger catheters, such as 10-Fr catheters, requires further investigation.

This study had some limitations. First, this was a retrospective observational study, and the choice of catheter diameter depended on the operator’s discretion. Differences in operator experience and catheter-size preference were not included in the PSM and may have influenced catheter selection and outcomes. Second, residual imbalances in fever and abdominal pain remained after PSM, whereas a residual imbalance in abdominal pain remained after IPTW. Residual or unmeasured confounding therefore cannot be excluded, particularly for clinical success. Third, clinical success was retrospectively assessed by investigators who were not blinded to catheter size, although predefined criteria were used. Fourth, the analyses of individual AEs and post-hoc subgroups were limited by small numbers of events and were not adjusted for multiple comparisons. Fifth, this was a single-center study evaluating only 6-Fr and 8-Fr catheters, which may limit generalizability. Finally, although 30-day follow-up was complete, subsequent management and follow-up duration were not standardized; therefore, the findings cannot be extrapolated to long-term catheter management.

## Conclusion

Compared with 6-Fr catheters, 8-Fr catheters were associated with fewer short-term AEs among patients undergoing PTGBD for acute calculous cholecystitis. The use of an 8-Fr catheter may also be associated with earlier achievement of clinical success, although clinical success rates were similar between the groups by day 14. These findings suggest that an 8-Fr catheter may be a reasonable option for short-term PTGBD management, depending on the clinical context.

## Supplementary Information

Supplementary Material 1.

## Data Availability

The datasets used and/or analyzed during the current study are available from the corresponding author on reasonable request. The data are not publicly available because they contain information that could compromise the privacy of research participants.

## Abbreviations

AE: Adverse event
ASA-PS: American Society of Anesthesiologists Physical Status
CI: Confidence interval
CKD: Chronic kidney disease
CT: Computed tomography
IPTW: Inverse probability of treatment weighting
PSM: Propensity score matching
PTGBD: Percutaneous transhepatic gallbladder drainage
RD: Risk difference
RR: Relative risk
SMD: Standardized mean difference
