# Supplementary material for: Short-term clinical outcomes of 6-Fr versus 8-Fr catheters for percutaneous transhepatic gallbladder drainage in acute calculous cholecystitis: a propensity score-matched analysis
Source: BMC Gastroenterol. 2026 Aug 6;26:726. doi: 10.1186/s12876-026-05210-9 (PMC13729407; doi:10.1186/s12876-026-05210-9)
Supplement: Supplementary file 1 [file 12876_2026_5210_MOESM1_ESM.pdf]

Supplementary Table 1. Subsequent clinical course after PTGBD in the propensity score-matched cohort

	6-Fr group (n=71)	8-Fr group (n=71)
Subsequent cholecystectomy, n (%)	22 (31.0)	27 (38.0)
PTGBD catheter maintained until cholecystectomy, n (%)	14 (19.7)	10 (14.1)
PTGBD catheter removed before cholecystectomy, n (%)	8 (11.3)	16 (22.5)
Catheter status at cholecystectomy unknown, n (%)	0 (0.0)	1 (1.4)
Time from PTGBD to cholecystectomy, days, median [IQR]	81 [43, 116] (n=21)	82.5 [55.3, 119.8] (n=26)
Conversion to EGBS, n (%)	9 (12.7)	9 (12.7)
Permanent PTGBD catheter placement, n (%)	3 (4.2)	4 (5.6)
PTGBD catheter removal without subsequent cholecystectomy or EGBS, n (%)	35 (49.3)	30 (42.3)
Death before catheter removal or definitive management, n (%)	2 (2.8)	1 (1.4)
PTGBD catheter indwelling duration, days, median [IQR]	16 [11, 31] (n=65)	17 [12, 27] (n=65)
Recurrent cholecystitis after catheter removal with the gallbladder in situ, n/N (%)	3/52 (5.8)	5/54 (9.3)

Recurrent cholecystitis was evaluated among patients whose PTGBD catheters were removed while the gallbladder remained in situ and for whom follow-up data were available.

EGBS, endoscopic gallbladder stenting; IQR, interquartile range; PSM, propensity score matching; PTGBD, percutaneous transhepatic gallbladder drainage.
